# Survey of *Rickettsia* species in hematophagous arthropods from endemic areas for Japanese spotted fever in China

**DOI:** 10.3389/fcimb.2024.1384284

**Published:** 2024-04-25

**Authors:** Junhua Tian, Jing Liu, Jin Liu, Miao Lu, Xiaomin Chen, Kun Li

**Affiliations:** ^1^ Affiliation of Disinfection and Vector Control, Wuhan Center for Disease Control and Prevention, Wuhan, Hubei, China; ^2^ Clinical Laboratory, Jiangxia Center for Disease Control and Prevention, Wuhan, Hubei, China; ^3^ National Institute for Communicable Disease Control and Prevention, Chinese Center for Disease Control and Prevention, Beijing, China

**Keywords:** mosquitoes, tabanids, ticks, Japanese spotted fever, *Candidatus* Rickettsia tabanidii, *Candidatus* Rickettsia xingshanensis, *Candidatus* Rickettsia hubeiensis

## Abstract

Japanese spotted fever (JSF) is caused by *Rickettsia japonica*, mainly vectored by hard ticks. However, whether *R. japonica* can be transmitted by other arthropods remains unknown. Moreover, it is of interest to investigate whether other *Rickettsia* species cause spotted fever in endemic areas. In this study, a survey of *Rickettsia* species was performed in hematophagous arthropods (mosquitoes, tabanids, and ticks) from endemic areas for JSF in Hubei Province, central China. The results showed that the diversity and prevalence of *Rickettsia* species in mosquitoes are low, suggesting that mosquitoes may not be the vector of zoonotic *Rickettsia* species. A novel *Rickettsia* species showed a high prevalence (16.31%, 23/141) in tabanids and was named “*Candidatus* Rickettsia tabanidii.” It is closely related to *Rickettsia* from fleas and mosquitoes; however, its pathogenicity in humans needs further investigation. Five *Rickettsia* species were identified in ticks. *Rickettsia japonica*, the agent of JSF, was detected only in *Haemaphysalis longicornis* and *Haemaphysalis hystricis*, suggesting that they may be the major vectors of *R. japonica*. Notably, two novel species were identified in *H. hystricis* ticks, one belonging to the spotted fever group and the other potentially belonging to the ancestral group. The latter one named “*Candidatus* Rickettsia hubeiensis” may provide valuable insight into the evolutionary history of *Rickettsia*.

## Introduction

Spotted fever is caused by the spotted fever group *Rickettsia* species, which are mainly vectored and transmitted by ticks. In China, Japanese spotted fever (JSF) caused by *Rickettsia japonica* is a common disease in eastern and central areas such as Zhejiang, Hubei, and Henan provinces (Lu et al., 2018; Li et al., 2019; Li and Liu, 2021; Gao et al., 2022; Zhou et al., 2023). The main manifestations of JSF are fever, erythema, and eschar (Noguchi et al., 2018). Occasionally, JSF leads to inflammation of the central nervous system or even death (Gao et al., 2022; Zhou et al., 2023). *Rickettsia japonica* is transmitted by hard ticks; it has been detected in multiple tick species such as *Haemaphysalis longicornis*, *Haemaphysalis hystricis*, *Haemaphysalis flava*, and *Rhipicephalus microplus* (Zhao et al., 2021). However, some studies indicate that several patients infected with JSF report no tick bite history (Li et al., 2019). Therefore, it is important to investigate whether JSF is transmitted by other hematophagous arthropods. It should be also noted that the high *R. japonica* seroprevalence in rural populations in some endemic areas (Li et al., 2018) may also result from immune cross-reaction with other spotted fever group *Rickettsia* circulating in these areas. However, the prevalence of *Rickettsia* species in many arthropods from these areas remains largely unknown. To date, only several studies investigated the *Rickettsia* species in mosquitoes in China (Guo et al., 2016; Zhang et al., 2016, 2019; Li et al., 2022; Lu et al., 2023), and some of those reported *Rickettsia bellii* and *Rickettsia monacensis* in endemic areas of JSF. Meanwhile, no *Rickettsia* species have been reported in tabanids in China. The goal of this study was to investigate the presence of *Rickettsia* species in hematophagous arthropods (mosquitoes, tabanids, and ticks) collected from endemic areas for JSF in Hubei Province.

## Methods

### Sample collection and DNA extraction

**Figure 1:**
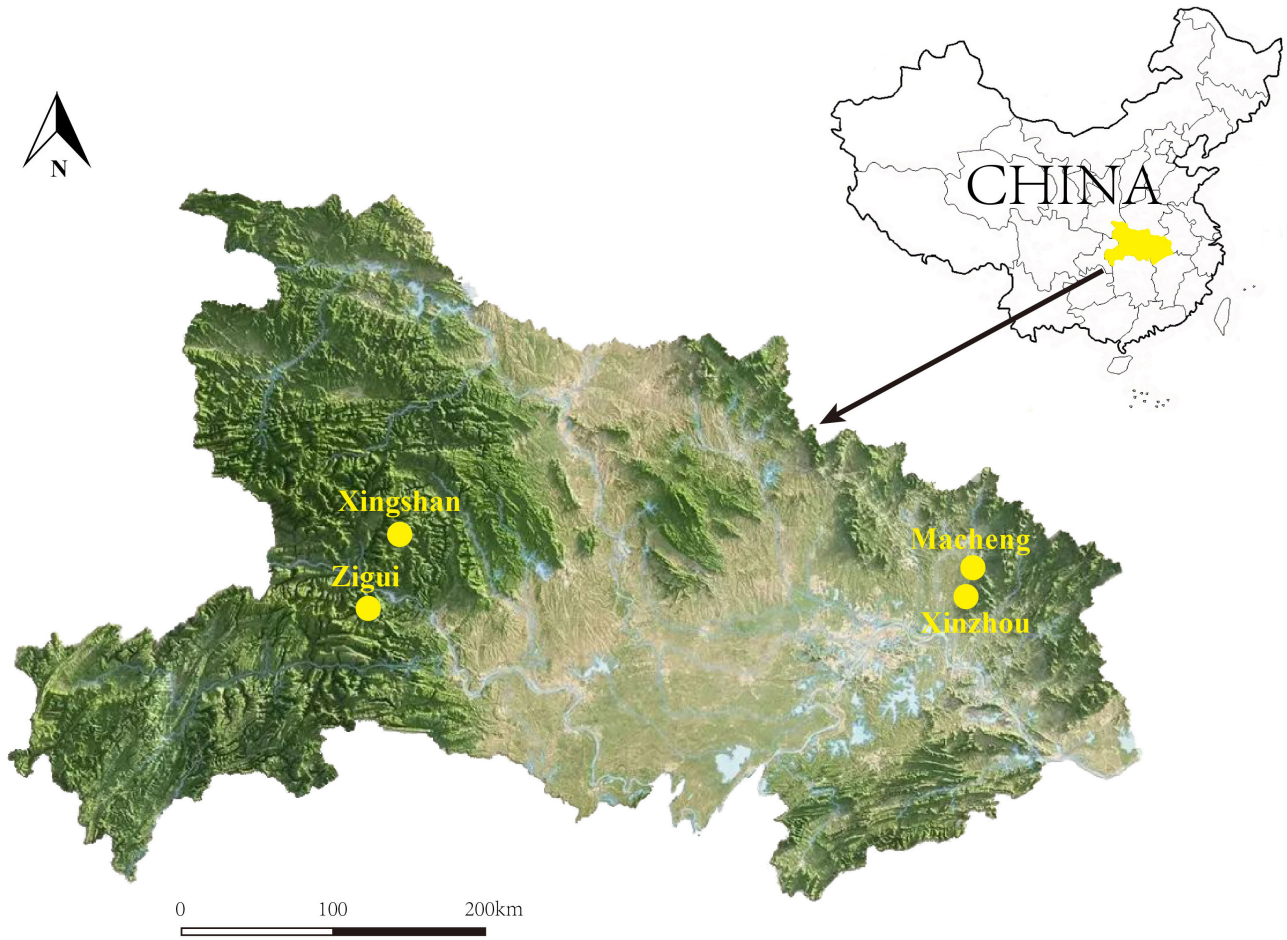
A map showing the locations of Zigui (110.98°E, 30.83°N), Macheng (115.01°E, 31.17°N), Xingshan (110.75°E, 31.35°N), and Xinzhou (114.80°E, 30.84°N) in Hubei Province, where the samples were collected.

In the summer and autumn of 2023, mosquitoes, blood-sucking flies, and ticks were collected from four endemic areas for JSF in Hubei Province: 1) Zigui County (110.98°E, 30.83°N) in Yichang City, 2) Xingshan County (110.75°E, 31.35°N) in Yichang City, 3) Macheng County (115.01°E, 31.17°N) in Huanggang City, and 4) Xinzhou District (114.80°E, 30.84°N) in Wuhan City (**Figure 1**). Mosquitoes were collected in hog pens, sheepfolds, and residential areas using a handheld mosquito trap (Li et al., 2022). Tabanids were collected in cowsheds using a handheld sweep net. We did not find suitable sampling areas for collecting insects in Xingshan, and there were no mosquito traps set in Macheng. Ticks were also collected from the bodies of domestic and roadkill animals opportunistically. All samples were frozen immediately in dry ice and then transported to the Wuhan Center for Disease Control and Prevention Laboratory, where morphological identification was performed (Teng and Jiang, 1991; Jiang et al., 2011; Changbunjong et al., 2021) and DNA was extracted. The entire specimen of mosquitoes and ticks was used for DNA extraction, while only the abdomen of tabanids was used. All samples were individually washed twice using phosphate-buffered saline and then homogenized in a mixer mill (Retsch MM400). DNA extraction was individually performed using the Omega Mollusc DNA kit.

### Detection of *Rickettsia* and amplification of key genes

All DNA samples were screened for *Rickettsia* species by hemi-nested PCR amplifying an approximately 900-bp sequence of the 16S rRNA gene. PCR products with the expected size were subjected to sequencing. The sequences were aligned with those in the GenBank Database using BLASTn (https://blast.ncbi.nlm.nih.gov) to identify the potential *Rickettsia* species. Subsequently, the *gltA* (citrate synthase), *groEL* (60 kDa chaperonin), and *ompB* (outer member protein B) partial gene sequences were amplified from *Rickettsia*-positive samples for further analysis. Additionally, the *ompA* gene was amplified from spotted fever group *Rickettsia* strains. All primers used are shown in **Table 1**.

**Table 1:** The primers used to amplify 16S, *gltA*, *groEL*, *ompA*, and *ompB* genes from *Rickettsia* strains by hemi-nested PCR.

Primer	Cycle	Gene	Sequence	Anticipated amplicon length
Ric-F	1, 2	16S	5-YTACGGAATAACTTTTAGAAA-3	900 bp
Ric-R1	1	16S	5-CATGATGACTTGACRTCGT-3	
Ric-R2	2	16S	5-CATCTCACGACACGAGCTG-3	
Ric-glt-F1	1	*gltA*	5-CCGGGYTTTATGTCTACTGC-3	1,100 bp
Ric-glt-F2	2	*gltA*	5-CTTTATGTCTACTGCKTCTTG-3	
Ric-glt-R	1, 2	*gltA*	5-AGCTGTCTWGGTCTGCTGATT-3	
Ric-gro-F1	1	*groEL*	5-CCATTACATGATAGAATTGCAAT-3	1,100 bp
Ric-gro-F2	2	*groEL*	5-GAATTGCAATAAAGCCTATCG-3	
Ric-gro-R	1, 2	*groEL*	5-CCATCATTGCTTTTCTTCTATC-3	
Rr190.70	1, 2	*ompA*	5-ATGGCGAATATTTCTCCAAA-3	700 bp
Ric-R1	1	*ompA*	5-ACCTACATTATCAAHGCCTGT-3	
Ric-R2	2	*ompA*	5-ACCTSTTAATACTGCATTTRCAT-3	
NompB-ex5	1	*ompB*	5-TAYTCCATYTTAGYATCWGRTCTTAT-3	550–650 bp
NompB-in5	2	*ompB*	5-STTSCACTTAAACCTARATTRTAAG-3	
NompB-3	1, 2	*ompB*	5-GTKTAGCWGGTTAYAAAGCTAAAAC-3	

### Sequence analysis

All the recovered sequences were sequenced in both directions. In addition, chimeras have been screened and removed. All sequences were compared with GenBank sequences using BLAST to determine nucleotide similarity. Sequences obtained in this study and reference sequences downloaded from the GenBank database were aligned using the MegAlign program in Lasergene (Burland, 2000). Maximum-likelihood (ML) trees were reconstructed using PhyML v3.0 (Guindon et al., 2009) under the best-fit substitution model determined by MEGA 7.0 (Kumar et al., 2016).

## Results

### Sample collections

Between July and October 2023, 527 adult mosquitoes, 141 adult tabanids, and 276 ticks (275 adult and 1 larva) were collected from four locations in Hubei Province. In Zigui County, 202 mosquitoes (48 *Culex tritaeniorhynchus*, 50 *Armigeres subalbatus*, 48 *Aedes albopictus*, 54 *Culex quinquefasciatus*, and 2 *Anopheles sinensis*), 67 tabanids (67 *Tabanus* spp.), and 196 ticks (88 *H. longicornis*, 58 *H. flava*, 49 *R. microplus*, and 1 *H. hystricis*) were collected (**Table 2**). In Macheng County, 325 mosquitoes (79 C*. tritaeniorhynchus*, 69 A*. albopictus*, 51 A*. sinensis*, 1 *Coquillettidia crassipes*, 3 *Mansonia uniformis*, 98 A*. subalbatus*, and 24 C*. quinquefasciatus*) were collected (**Table 2**). Additionally, 80 *H. hystricis* ticks were collected in Xingshan County, and 74 *Tabanus* spp. specimens were collected in Xinzhou District (**Table 2**). All species were morphologically identified by a trained taxonomist; however, tabanids were not identified at the species level. We tried to molecularly confirm the species of the tabanids, but the results were not decisive.

**Table 2:** Detection of *Rickettsia* spp. in hematophagous vector species from different sampling locations in Hubei Province, central China.

	Hematophagous arthropods	Locations	Number	*Rickettsia* species and positive rates
Mosquitoes	*Aedes albopictus*	Zigui	48 (F)	NA
		Macheng	69 (F)	NA
	*Anopheles sinensis*	Zigui	2 (F)	NA
		Macheng	51 (F)	NA
	*Armigeres subalbatus*	Zigui	50 (F)	NA
		Macheng	98 (F)	NA
	*Coquillettidia crassipes*	Macheng	1 (F)	NA
	*Culex quinquefasciatus*	Zigui	54 (F)	*R. bellii* (1.85%, 1/54)
		Macheng	24 (F)	NA
	*Culex tritaeniorhynchus*	Zigui	48 (F)	NA
		Macheng	79 (F)	*R. bellii* (1.27%, 1/79) *Ca.* Rickettsia jingxinensis (1.27%, 1/79)
	*Mansonia uniformis*	Macheng	3 (F)	NA
Tabanid	*Tabanus* spp.	Zigui	67	*Ca*. Rickettsia tabanidii (8.96%, 6/67)
		Xinzhou	74	*Ca*. Rickettsia tabanidii (22.97%, 17/74)
Ticks	*Haemaphysalis flava*	Zigui	17 (F) + 40 (M) + 1 (L)	*Rickettsia* sp. (5.17%, 3/58)
	*Haemaphysalis hystricis*	Zigui	1 (M)	NA
		Xingshan	53 (F) + 27 (M)	*Ca*. Rickettsia hubeiensis (3.75%, 3/80) *Ca*. Rickettsia xingshanensis (2.50%, 2/80) *R. japonica* (1.25%, 1/80)
	*Haemaphysalis longicornis*	Zigui	79 (F) + 9 (M)	*R. japonica* (1.14%, 1/88) *Ca.* Rickettsia jingxinensis (2.27%, 2/88)
	*Rhipicephalus microplus*	Zigui	35 (F) + 14 (M)	*Ca.* Rickettsia jingxinensis (14.29%, 7/49)

F, female; M, male; L, larva; NA, not available.

### *Rickettsia* spp. in mosquitoes and tabanids

Only three mosquitoes (0.57%, 3/527) tested positive for *Rickettsia* species. *Rickettsia bellii* was detected in one *C. tritaeniorhynchus* (strain MCW67) from Macheng and one *C. quinquefasciatus* (strain ZGCQ1) from Zigui, respectively. The *gltA* and *groEL* sequences of the strain ZGCQ1 showed 98.73% and 96.04% nucleotide identity with those of other *R. bellii* strains. Moreover, *Ca.* Rickettsia jingxinensis was detected in one *C. tritaeniorhynchus* specimen.

**Figure 2:**
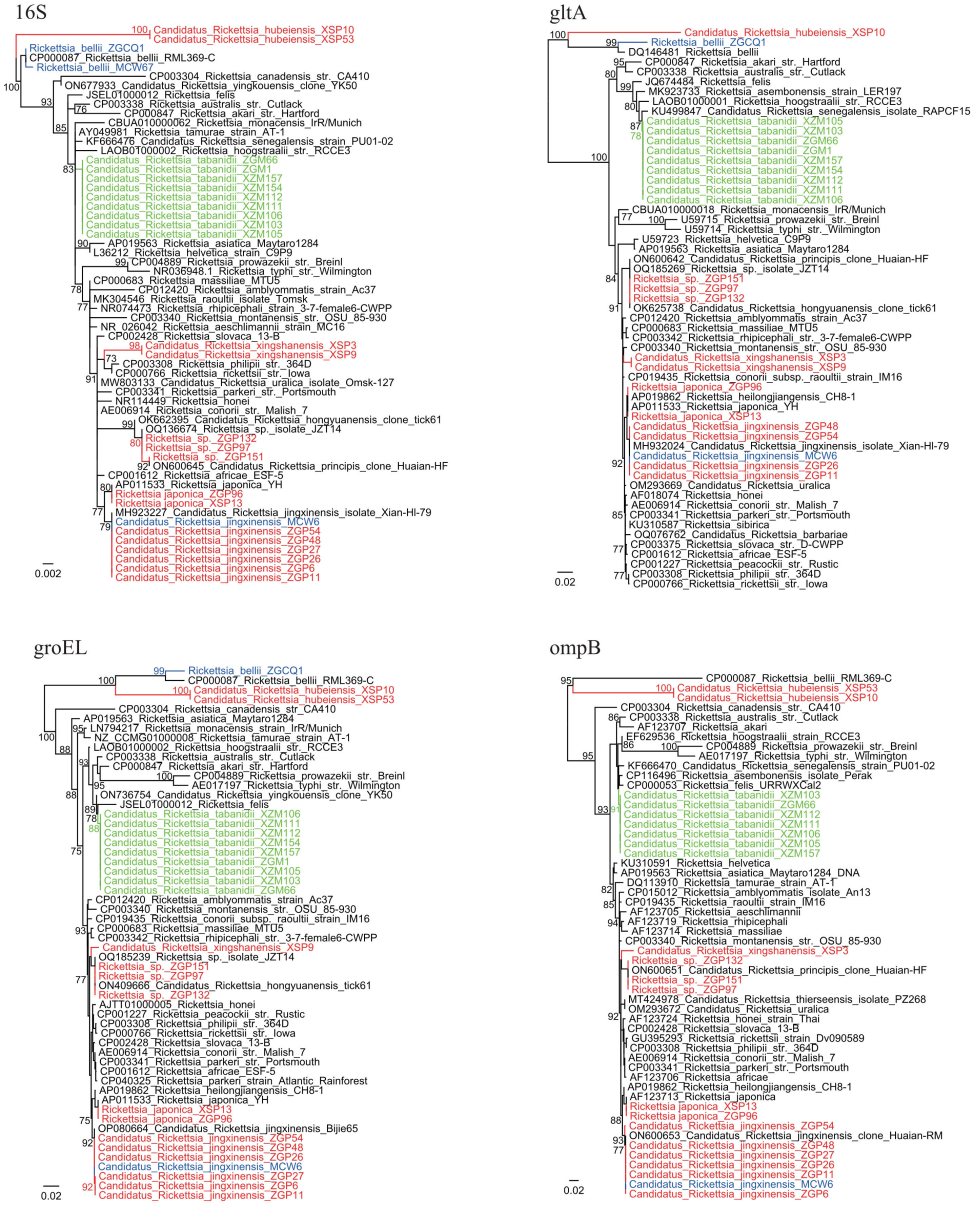
Phylogenetic trees based on the 16S (809 bp), *gltA* (792–947 bp), *groEL* (1,032–1,036 bp), and *ompB* (523–600 bp) sequences of the *Rickettsia* strains. Green: *Rickettsia* strains from tabanids. Blue: *Rickettsia* strains from mosquitoes. Red: *Rickettsia* strains from ticks.

Notably, tabanids from both locations were positive for a putative novel *Rickettsia* species (8.96% and 22.97% tabanids from Zigui and Macheng, respectively). Their 16S rRNA, *gltA*, *groEL*, and *ompB* sequences were identical between samples from the two locations despite the geographic distance. Genomic analysis showed that the 16S rRNA sequences had the highest identity (99.88%) with *Rickettsia tamurae* from ticks and *Rickettsia* endosymbiont of *Curculio* spp. The *gltA* sequences showed 99.14% similarity with *Candidatus* Rickettsia senegalensis identified in *Ctenocephalides felis* (fleas), whereas the *groEL* sequences were highly homologous (99.51%) to *Candidatus* Rickettsia yingkouensis previously identified in *A. sinensis* (mosquitoes) (Lu et al., 2023). The *ompB* sequences showed 98.83% similarity with *Rickettsia asembonensis* strains identified in *C. canis* (fleas). In the phylogenetic trees (**Figure 2**), these strains were clustered in an independent clade. According to the criteria established by Fournier et al. (2003), a novel *Rickettsia* species should have at most one of the following sequence similarity degrees when compared with validated *Rickettsia* species: 16S rDNA ≥99.8%, *gltA* genes ≥99.9%, and, when available, ≥98.8%, ≥99.2%, and ≥99.3% for the *ompA*, *ompB*, and *sca4* genes, respectively. These results may propose that it represents a putative novel species. Herein, we named it “*Candidatus* Rickettsia tabanidii.”

### *Rickettsia* spp. in ticks

Five *Rickettsia* species were identified in ticks. Only *Ca.* Rickettsia jingxinensis (14.29%, 7/49) was detected in *R. microplus* ticks. Similarly, only one *Rickettsia* species closely related to *Ca.* Rickettsia hongyuanensis and *Ca*. Rickettsia principis (5.17%, 3/58) was detected in *H. flava* ticks. Phylogenetic analysis of 16S rRNA and *ompA* sequences indicated certain genetic variations among these strains (**Figure 2**, **Supplementary Figure S1**). In *H. longicornis* ticks, *R. japonica*, the causative agent of JSF (1.14%, 1/88), as well as *Ca.* Rickettsia jingxinensis (2.27%, 2/88), was detected. Notably, three *Rickettsia* species were identified in *H. hystricis* ticks: *R. japonica* (1.25%, 1/80) and two novel species (**Table 2**). For one of these species (strains XSP3 and XSP9), the 16S rRNA sequences were 100% identical to uncultured *Rickettsia* sp. Hhy_2024 in *H. hystricis* ticks from Japan and 99.38% identical to *Rickettsia peacockii* str. Rustic. Moreover, the *gltA* sequences were 100% identical to *Rickettsia* sp. MT55-R identified in *H. hystricis* ticks from Japan and 98.73% identical to *R. raoultii* strains. The *groEL* and *ompB* sequences were also amplified and showed the highest nucleotide similarity to *Ca.* Rickettsia hongyuanensis tick61 (98.55%) and *Rickettsia* sp. strain RH-9699 (96.83%), respectively. According to the criteria established by Fournier et al. (2003), although its *ompA* gene was not amplifiable, our data suggested that it was a novel species belonging to the spotted fever group, and it was named “*Candidatus* Rickettsia xingshanensis.”

Notably, another novel *Rickettsia* species (strains XSP10, XSP53, and XSP76) located at the base of the phylogenetic trees was characterized and named “*Candidatus* Rickettsia hubeiensis” (**Figure 2**). The 16S rRNA sequences showed the highest identity (98.49%) with *Rickettsia honei* and 97.89% identity with *R. bellii*. The *gltA*, *groEL*, and *ompB* sequences were all successfully recovered, showing a nucleotide identity of 92.81% with *Rickettsia* endosymbiont of *Amblyomma patinoi*, 89.45% with *R. bellii*, and 79.09% with *R. asembonensis* (93% coverage), respectively. Notably, its *ompB* gene appeared to be truncated. In the phylogenetic trees, this species formed a sister clade with the ancestral *Rickettsia* species *R. bellii*.

## Discussion

In this study, we investigated whether *Rickettsia* species were harbored by hematophagous arthropods (mosquitoes, tabanids, and ticks) collected from endemic areas for JSF in China. A total of seven *Rickettsia* species were identified, including *R. japonica*—the causative agent of JSF—and three putative novel *Rickettsia* species. Of the studied arthropods, ticks, especially *H. hystricis* ticks, were demonstrated to harbor the most diverse *Rickettsia* species.

Previous studies have suggested that mosquitoes harbor several *Rickettsia* species belonging to various groups, and the role of mosquitoes in the transmission of *Rickettsia* has been speculated (Guo et al., 2016; Zhang et al., 2019; Pollio et al., 2022). However, the present study identified only two *Rickettsia* species in mosquitoes. It is surprising that *Ca.* Rickettsia jingxinensis, a spotted fever group *Rickettsia* frequently detected in ticks (Guo et al., 2019; Wang et al., 2021; Lu et al., 2022), was identified in one *C. tritaeniorhynchus* sample. Natural infection of mosquitoes with *Ca.* Rickettsia jingxinensis is not likely. In addition, the *Ca.* Rickettsia jingxinensis-positive mosquito sample was engorged. Therefore, we speculate that the finding is attributable to a blood meal that the mosquitoes derived from a natural animal host of *Ca.* Rickettsia jingxinensis. In previous studies, the positive rate of *R. belli* in mosquitoes may vary from 0.52% to 24.49% (Guo et al., 2016; Li et al., 2022). Although some serological investigations showed that animals (dogs, horses, etc.) may be exposed to *R. bellii* (de Oliveira et al., 2019; Neves et al., 2020), no infection case has been reported in animals or humans. In other words, there is no concrete evidence for the pathogenicity of these two *Rickettsia* species. Additionally, the positive rates of both *Ca.* Rickettsia jingxinensis and *R. belli* were extremely low in mosquitoes. Therefore, we speculate that mosquitoes do not act as the vector of zoonotic *Rickettsia* species in this area.

It is out of our expectation that tabanids harbor a novel *Rickettsia* species. Compared with the positive rate reported by Keita et al. (5.1%), the prevalence of *Ca.* Rickettsia tabanidii in tabanids is quite high (16.31%, 23/141). Although it is well known that tabanids bite humans, tabanids have long been overlooked as a potential vector of zoonotic pathogens. In 2020, Keita et al. detected *Rickettsia africae*, *Rickettsia slovaca*, and *Rickettsia montanensis* in the tabanid species *Atylotus fuscipes* from Senegal, West Africa (Keita et al., 2020). To our knowledge, this is the only report to date of tabanids harboring *Rickettsia* species. In the present study, the high prevalence of *Ca*. Rickettsia tabanidii in samples from two locations suggests that it might have a broad distribution and *Rickettsia* species could be more frequently carried by tabanids than is reported. More attention should be paid to its human pathogenicity and the associated threat to public health.

Five *Rickettsia* species were identified in ticks. *Rickettsia japonica*, the agent of JSF, was detected only in *H. longicornis* and *H. hystricis* ticks, suggesting that these two tick species may be the main vectors of *R. japonica*. This finding is consistent with previous reports (Zhao et al., 2021). Moreover, *Candidatus* Rickettsia jingxinensis, also named *Candidatus* Rickettsia longicornii (Guo et al., 2019), was detected in *H. longicornis* and *R. microplus* ticks from Zigui County. This finding was not unexpected because it is a widely distributed spotted fever group *Rickettsia* species and has previously been detected in *R. microplus* and *H. longicornis* ticks from multiple provinces in China (Guo et al., 2019; Wang et al., 2021; Lu et al., 2022). Additionally, two spotted fever group *Rickettsia* species—*Ca.* Rickettsia xingshanensis and an unidentified *Rickettsia* closely related to both *Ca.* Rickettsia hongyuanensis and *Ca*. Rickettsia principis—were identified in *H. hystricis* and *H. flava*, respectively. Although there is still no evidence that they infect humans, their close relationship with pathogenic species suggests a potential risk. Both *H. hystricis* and *H. flava* have been reported to bite humans (Grassman et al., 2004; Kim et al., 2022), which potentially increases the risk. Therefore, it is necessary to investigate whether these *Rickettsia* species are the causative agents of spotted fever in patients in this area.

In *H. hystricis* ticks, *Ca.* Rickettsia hubeiensis, a putative novel *Rickettsia* species located in the ancestral position in the phylogenetic trees, was identified. On the basis of phylogenomic analysis, the genus *Rickettsia* can be separated into five groups: two spotted fever groups, a typhus group, a canadensis group, and an ancestral bellii group (El Karkouri et al., 2022). The bellii group contains *R. bellii*, and it is the earliest diverging group among the genus *Rickettsia*. In phylogenetic trees based on sequences obtained in this study, the *Ca.* Rickettsia hubeiensis strains formed a sister clade with *R. bellii* and formed the outgroup for all other *Rickettsia* members. Although its genome sequences remain unavailable, we propose that it may be a novel member of the bellii group. The study of its genome sequence may elucidate the evolutionary history of *Rickettsia*.

## Data availability statement

The datasets presented in this study can be found in online repositories. The names of the repository/repositories and accession number(s) can be found in the article/**Supplementary Material**.

## Ethics statement

The manuscript presents research on animals that do not require ethical approval for their study.

## Author contributions

JT: Writing – review & editing, Resources, Investigation, Funding acquisition. JingL: Writing – review & editing, Investigation. JinL: Writing – review & editing. ML: Writing – review & editing, Methodology. XC: Writing – review & editing, Investigation. KL: Writing – review & editing, Writing – original draft, Conceptualization.

## Supplementary material

Supplementary Figure S1 Phylogenetic tree based on the *ompA* (677-696 bp) sequences of the spotted fever group *Rickettsia* strains. Blue: *Rickettsia* strains from mosquitoes. Red: *Rickettsia* strains from ticks.
