# Supplementary figures and images for: Survey of Rickettsia species in hematophagous arthropods from endemic areas for Japanese spotted fever in China
Source: Front Cell Infect Microbiol. 2024 Apr 25;14:1384284. doi: 10.3389/fcimb.2024.1384284 (PMC11079133; doi:10.3389/fcimb.2024.1384284)

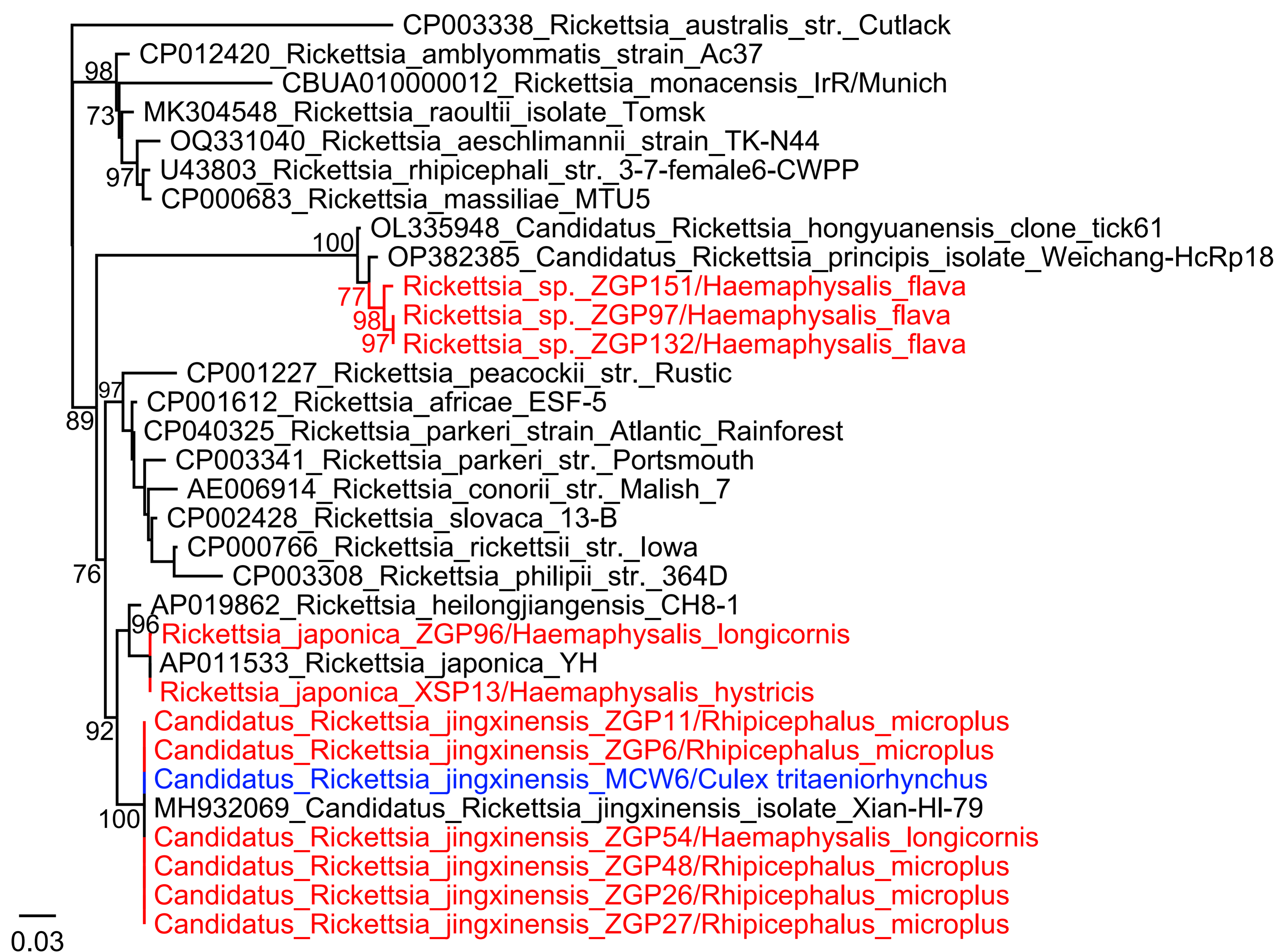

Supplement: Supplementary Figure S1 — Phylogenetic tree based on the ompA (677-696 bp) sequences of the spotted fever group Rickettsia strains. Blue: Rickettsia strains from mosquitoes. Red: Rickettsia strains from ticks. [file Image_1.pdf]
