# Supplementary material for: Survey of Rickettsia species in hematophagous arthropods from endemic areas for Japanese spotted fever in China
Source: Front Cell Infect Microbiol. 2024 Apr 25;14:1384284. doi: 10.3389/fcimb.2024.1384284 (PMC11079133; doi:10.3389/fcimb.2024.1384284)
Supplement: Supplementary file 2 [file Table_1.docx]

Table S1. Genbank numbers of the *Rickettsia* sequences obtained in this study.

|  | **Gene** | **Genbank numbers** | **Bacterial strain** |
| --- | --- | --- | --- |
| 1 | 16S | OR979071 | Candidatus_Rickettsia_jingxinensis_ZGP6 |
| 2 | 16S | OR979072 | Candidatus_Rickettsia_jingxinensis_ZGP11 |
| 3 | 16S | OR979073 | Candidatus_Rickettsia_jingxinensis_ZGP26 |
| 4 | 16S | OR979074 | Candidatus_Rickettsia_jingxinensis_ZGP27 |
| 5 | 16S | OR979075 | Candidatus_Rickettsia_jingxinensis_ZGP48 |
| 6 | 16S | OR979076 | Candidatus_Rickettsia_jingxinensis_ZGP54 |
| 7 | 16S | OR979077 | Rickettsia japonica_ZGP96 |
| 8 | 16S | OR979078 | Rickettsia japonica_XSP13 |
| 9 | 16S | OR979079 | Rickettsia_sp._ZGP97 |
| 10 | 16S | OR979080 | Rickettsia_sp._ZGP132 |
| 11 | 16S | OR979081 | Rickettsia_sp._ZGP151 |
| 12 | 16S | OR979082 | Candidatus_Rickettsia_hubeiensis_XSP10 |
| 13 | 16S | OR979083 | Candidatus_Rickettsia_hubeiensis_XSP53 |
| 14 | 16S | OR979084 | Candidatus_Rickettsia_xingshanensis_XSP3 |
| 15 | 16S | OR979085 | Candidatus_Rickettsia_xingshanensis_XSP9 |
| 16 | 16S | OR979086 | Rickettsia_bellii_MCW67 |
| 17 | 16S | OR979087 | Candidatus_Rickettsia_jingxinensis_MCW6 |
| 18 | 16S | OR979088 | Rickettsia_bellii_ZGCQ1 |
| 19 | 16S | OR979089 | Candidatus_Rickettsia_tabanidii_XZM103 |
| 20 | 16S | OR979090 | Candidatus_Rickettsia_tabanidii_XZM105 |
| 21 | 16S | OR979091 | Candidatus_Rickettsia_tabanidii_XZM106 |
| 22 | 16S | OR979092 | Candidatus_Rickettsia_tabanidii_XZM111 |
| 23 | 16S | OR979093 | Candidatus_Rickettsia_tabanidii_XZM112 |
| 24 | 16S | OR979094 | Candidatus_Rickettsia_tabanidii_XZM154 |
| 25 | 16S | OR979095 | Candidatus_Rickettsia_tabanidii_XZM157 |
| 26 | 16S | OR979096 | Candidatus_Rickettsia_tabanidii_ZGM1 |
| 27 | 16S | OR979097 | Candidatus_Rickettsia_tabanidii_ZGM66 |
| 28 | groEL | OR971698 | Rickettsia_bellii_ZGCQ1 |
| 29 | groEL | OR971699 | Candidatus_Rickettsia_jingxinensis_MCW6 |
| 30 | groEL | OR971700 | Candidatus_Rickettsia_tabanidii_XZM103 |
| 31 | groEL | OR971701 | Candidatus_Rickettsia_tabanidii_XZM105 |
| 32 | groEL | OR971702 | Candidatus_Rickettsia_tabanidii_XZM106 |
| 33 | groEL | OR971703 | Candidatus_Rickettsia_tabanidii_XZM111 |
| 34 | groEL | OR971704 | Candidatus_Rickettsia_tabanidii_XZM112 |
| 35 | groEL | OR971705 | Candidatus_Rickettsia_tabanidii_XZM154 |
| 36 | groEL | OR971706 | Candidatus_Rickettsia_tabanidii_XZM157 |
| 37 | groEL | OR971707 | Candidatus_Rickettsia_tabanidii_ZGM1 |
| 38 | groEL | OR971708 | Candidatus_Rickettsia_tabanidii_ZGM66 |
| 39 | groEL | OR971709 | Candidatus_Rickettsia_jingxinensis_ZGP6 |
| 40 | groEL | OR971710 | Candidatus_Rickettsia_jingxinensis_ZGP11 |
| 41 | groEL | OR971711 | Candidatus_Rickettsia_jingxinensis_ZGP26 |
| 42 | groEL | OR971712 | Candidatus_Rickettsia_jingxinensis_ZGP27 |
| 43 | groEL | OR971713 | Candidatus_Rickettsia_jingxinensis_ZGP48 |
| 44 | groEL | OR971714 | Candidatus_Rickettsia_jingxinensis_ZGP54 |
| 45 | groEL | OR971715 | Rickettsia japonica_ZGP96 |
| 46 | groEL | OR971716 | Rickettsia_sp._ZGP97 |
| 47 | groEL | OR971717 | Rickettsia_sp._ZGP132 |
| 48 | groEL | OR971718 | Rickettsia_sp._ZGP151 |
| 49 | groEL | OR971719 | Candidatus_Rickettsia_xingshanensis_XSP9 |
| 50 | groEL | OR971720 | Candidatus_Rickettsia_hubeiensis_XSP10 |
| 51 | groEL | OR971721 | Candidatus_Rickettsia_hubeiensis_XSP53 |
| 52 | groEL | OR971722 | Rickettsia japonica_XSP13 |
| 53 | gltA | OR971676 | Candidatus_Rickettsia_jingxinensis_MCW6 |
| 54 | gltA | OR971678 | Rickettsia_bellii_ZGCQ1 |
| 55 | gltA | OR971679 | Candidatus_Rickettsia_tabanidii_XZM103 |
| 56 | gltA | OR971680 | Candidatus_Rickettsia_tabanidii_XZM105 |
| 57 | gltA | OR971681 | Candidatus_Rickettsia_tabanidii_XZM106 |
| 58 | gltA | OR971682 | Candidatus_Rickettsia_tabanidii_XZM111 |
| 59 | gltA | OR971683 | Candidatus_Rickettsia_tabanidii_XZM112 |
| 60 | gltA | OR971684 | Candidatus_Rickettsia_tabanidii_XZM154 |
| 61 | gltA | OR971685 | Candidatus_Rickettsia_tabanidii_XZM157 |
| 62 | gltA | OR971686 | Candidatus_Rickettsia_tabanidii_ZGM1 |
| 63 | gltA | OR971687 | Candidatus_Rickettsia_tabanidii_ZGM66 |
| 64 | gltA | OR971688 | Candidatus_Rickettsia_jingxinensis_ZGP11 |
| 65 | gltA | OR971689 | Candidatus_Rickettsia_jingxinensis_ZGP26 |
| 66 | gltA | OR971690 | Candidatus_Rickettsia_xingshanensis_XSP3 |
| 67 | gltA | OR971691 | Candidatus_Rickettsia_jingxinensis_ZGP48 |
| 68 | gltA | OR971692 | Candidatus_Rickettsia_jingxinensis_ZGP54 |
| 69 | gltA | OR971693 | Rickettsia japonica_XSP13 |
| 70 | gltA | OR971694 | Rickettsia japonica_ZGP96 |
| 71 | gltA | OR971695 | Rickettsia_sp._ZGP97 |
| 72 | gltA | OR971696 | Rickettsia_sp._ZGP132 |
| 73 | gltA | OR971697 | Rickettsia_sp._ZGP151 |
| 74 | ompA | OR995260 | Candidatus_Rickettsia_jingxinensis_MCW6 |
| 75 | ompA | OR995261 | Candidatus_Rickettsia_jingxinensis_ZGP6 |
| 76 | ompA | OR995262 | Candidatus_Rickettsia_jingxinensis_ZGP11 |
| 77 | ompA | OR995263 | Candidatus_Rickettsia_jingxinensis_ZGP26 |
| 78 | ompA | OR995264 | Candidatus_Rickettsia_jingxinensis_ZGP27 |
| 79 | ompA | OR995265 | Candidatus_Rickettsia_jingxinensis_ZGP48 |
| 80 | ompA | OR995266 | Candidatus_Rickettsia_jingxinensis_ZGP54 |
| 81 | ompA | OR995267 | Rickettsia japonica_ZGP96 |
| 82 | ompA | OR995268 | Rickettsia japonica_XSP13 |
| 83 | ompA | OR995269 | Rickettsia_sp._ZGP97 |
| 84 | ompA | OR995270 | Rickettsia_sp._ZGP132 |
| 85 | ompA | OR995271 | Rickettsia_sp._ZGP151 |
| 86 | ompB | PP146537 | Candidatus_Rickettsia_hubeiensis_XSP53 |
| 87 | ompB | PP146538 | Candidatus_Rickettsia_hubeiensis_XSP10 |
| 88 | ompB | PP146539 | Candidatus_Rickettsia_jingxinensis_MCW6 |
| 89 | ompB | PP146540 | Candidatus_Rickettsia_xingshanensis_XSP3 |
| 90 | ompB | PP146541 | Rickettsia japonica_XSP13 |
| 91 | ompB | PP146542 | Candidatus_Rickettsia_tabanidii_XZM103 |
| 92 | ompB | PP146543 | Candidatus_Rickettsia_tabanidii_XZM105 |
| 93 | ompB | PP146544 | Candidatus_Rickettsia_tabanidii_XZM106 |
| 94 | ompB | PP146545 | Candidatus_Rickettsia_tabanidii_XZM111 |
| 95 | ompB | PP146546 | Candidatus_Rickettsia_tabanidii_XZM112 |
| 96 | ompB | PP146547 | Candidatus_Rickettsia_tabanidii_XZM157 |
| 97 | ompB | PP146548 | Candidatus_Rickettsia_tabanidii_ZGM66 |
| 98 | ompB | PP146549 | Candidatus_Rickettsia_jingxinensis_ZGP6 |
| 99 | ompB | PP146550 | Candidatus_Rickettsia_jingxinensis_ZGP11 |
| 100 | ompB | PP146551 | Candidatus_Rickettsia_jingxinensis_ZGP26 |
| 101 | ompB | PP146552 | Candidatus_Rickettsia_jingxinensis_ZGP27 |
| 102 | ompB | PP146553 | Candidatus_Rickettsia_jingxinensis_ZGP48 |
| 103 | ompB | PP146554 | Candidatus_Rickettsia_jingxinensis_ZGP54 |
| 104 | ompB | PP146555 | Rickettsia japonica_ZGP96 |
| 105 | ompB | PP146556 | Rickettsia_sp._ZGP97 |
| 106 | ompB | PP146557 | Rickettsia_sp._ZGP132 |
| 107 | ompB | PP146558 | Rickettsia_sp._ZGP151 |
